# Evaluation of the Symptoms and Clinical Characteristics of Crohn’s Disease and Ulcerative Colitis That Affect Healthcare Providers’ Treatment Choices

**DOI:** 10.1093/crocol/otae053

**Published:** 2024-10-05

**Authors:** Theresa Hunter Gibble, Carolyn Sweeney, Daniel Wolin, David McSorley, Jinyi Wang, Richard Moses, Marla Dubinsky

**Affiliations:** Eli Lilly and Company, Medical Affairs, Indianapolis, IN, USA; RTI Health Solutions, Research Triangle Park, NC, USA; RTI Health Solutions, Research Triangle Park, NC, USA; RTI Health Solutions, Research Triangle Park, NC, USA; RTI Health Solutions, Research Triangle Park, NC, USA; Eli Lilly and Company, Medical Affairs, Indianapolis, IN, USA; Division of Pediatric Gastroenterology, Icahn School of Medicine, Mount Sinai, New York City, NY, USA

**Keywords:** inflammatory bowel disease (IBD), treatment choices, IBD symptoms

## Abstract

**Background:**

Treatment of inflammatory bowel disease—Crohn’s disease (CD) and ulcerative colitis (UC)—is dependent on healthcare providers’ (HCPs’) clinical assessment of patient symptoms. We therefore evaluated which CD and UC symptoms impact HCPs’ treatment choices and assessed the impact of those symptoms on treatment decision-making. We also examined the role of complete control (mucosal/histologic healing, clinical remission, no bowel urgency) in treatment decision-making, considerations for dose escalation or switching treatments, and HCPs’ willingness to use the Urgency Numeric Rating Scale (NRS) to assess bowel urgency severity.

**Methods:**

We conducted an observational, cross-sectional, self-administered survey among HCPs (*N* = 459, across types/specialties) who work in direct patient care and treat patients with CD and UC in the United States. Data were collected from eligible participants between November 21, 2022, and December 6, 2022, and responses were summarized through descriptive statistics.

**Results:**

For CD and UC, the symptoms of greatest importance when deciding on the course of treatment included cramping or abdominal pain, rectal bleeding, diarrhea, anemia, weight loss, and bowel urgency. Furthermore, most HCPs ranked rectal bleeding, clinical remission, abdominal pain, and complete control as “very” to “extremely” important in decisions about the course of treatment, dose escalation, or switching treatments. In total, 22.9% of HCPs indicated that they use the Urgency NRS, while 89.3% were at least somewhat willing to use it in the future.

**Conclusions:**

Our study provides real-world insights into the symptoms and clinical characteristics that most impact HCPs’ treatment choices for CD and UC in clinical practice.

## Introduction

Inflammatory bowel disease (IBD), including both Crohn’s disease (CD) and ulcerative colitis (UC), is characterized by chronic inflammation of the gastrointestinal tract and is associated with widespread morbidity.^1^ Over 6.8 million patients have been diagnosed with IBD worldwide, and the prevalence of cases continues to increase globally.^2,3^ Symptoms of IBD vary across patients but commonly include bowel urgency, persistent diarrhea, abdominal pain, rectal bleeding, weight loss, and fatigue.^4,5^ Patients with IBD often experience changes in symptoms and disease activity, alternating between periods of clinical remission (having reduced or no symptoms) and active disease (having symptoms or “flares”),^5^ although disease activity does not guarantee a correlation with disease severity.^6^

Clinical guidelines recommend approaches to treat CD^7^ and UC.^8^ However, symptoms of IBD are heterogeneous, and treatment often requires a more tailored approach.^5^ Common treatments for CD include immunomodulators, biologics, small molecules—like upadacitinib, which was approved to treat CD in the United States^9^ in 2023 (after this study)—and corticosteroids,^10^ while common treatments for UC include mesalamine therapies, immunomodulators, biologics, small molecules, and corticosteroids.^11^ Unfortunately, medications do not always control disease activity, and patients with CD or UC complications may eventually require surgery.^5^ Overall, the main treatment goals for patients with IBD are to reduce or resolve the signs and symptoms of active disease and to promote mucosal healing.^7,8,12,13^ Effective IBD treatment also includes a “treat-to-target” approach, which involves proactive monitoring of symptoms and disease activity to guide treatment decisions and improve long-term outcomes.^14^

Despite the need for tailored approaches to treat patients with CD and UC,^5^ little is known about IBD symptoms and characteristics that factor into healthcare providers’ (HCPs’) treatment decision-making. Therefore, the current study was designed to (1) provide insight into the symptoms and clinical characteristics that most factor into HCPs’ treatment choices for their patients with CD and UC in a real-world clinical setting and (2) assess HCPs’ willingness to use the Urgency Numeric Rating Scale (Urgency NRS, a validated patient-reported outcome measure)^15,16^ to evaluate the severity of bowel urgency. The results of this study may provide insight into the symptoms and clinical characteristics that impact HCP treatment choices, thereby informing future interventions and ensuring optimal outcomes for patients with CD and UC.

## Methods

### Study Design

We conducted an observational, cross-sectional, web-based survey among a sample of HCPs who treat patients with CD and UC in the United States. The primary objectives were to determine which symptoms of CD and UC affect HCPs’ treatment choices and to evaluate the importance of specific factors (ie, bowel urgency, stool frequency, abdominal pain, rectal bleeding, fatigue, gastrointestinal mucosal and histologic appearance, and clinical remission) on HCPs’ treatment choices for patients with CD or UC. Additionally, we aimed to explore the role of complete control (mucosal/histologic healing, clinical remission, and no bowel urgency) in treatment decision-making, identify HCPs’ decision-making considerations when escalating the dose of a patient’s current treatment or switching them to a different advanced therapy, and evaluate HCPs’ willingness to use the Urgency NRS to assess the severity of bowel urgency in their clinical practice.

Treatment of IBD often involves an integrated team of various HCP types and specialties.^17,18^ Thus, our study included HCPs currently practicing in the United States as gastroenterologists (GI), family medicine physicians (FM) internal medicine physicians (IM), primary care physicians (PCP) in family medicine or internal medicine, nurse practitioners (NP), or physician assistant (PA). To be eligible, HCPs had to work an average of >20 hours per week in direct patient care and treat an average of ≥10 patients with CD and ≥10 patients with UC (all adults, aged ≥18 years) per month. Eligible HCPs also had to be able to complete the study survey in English. Study participants were recruited by selecting a simple random sample of HCPs through an online partner specializing in recruitment services. Recruitment targets were up to 450 HCPs across the following types and specialties: GIs (*n* = 100), FM/IM/PCPs (*n* = 150), NPs (*n* = 100), and PAs (*n* = 100).

### Survey Development and Cognitive Pretesting

Questionnaire items were drafted using standard survey methodological principles.^19^ Survey questions were developed to elicit information on the characteristics of study participants and their clinical practice—including HCPs’ current use of and willingness to use the Urgency NRS—and to address the other study objectives. The Urgency NRS is a valid and reliable patient-reported outcome measure used to evaluate the severity of bowel urgency in patients with CD and UC, where patients report bowel urgency in the past 24 hours using a single 11-point Urgency NRS ranging from 0 to 10 (“No Urgency” to “Worst Possible Urgency”).^15,16^

Cognitive pretesting was conducted to evaluate the draft survey questionnaire and ensure that the items, response options, and recall periods would be understandable and easily answered by survey participants. HCPs (1 GI, 2 PCPs, 1 NP, and 1 PA) were recruited for cognitive pretesting using the same eligibility criteria as the full sample, to ensure that participant demographics and characteristics were representative of the target population. After HCPs provided verbal consent to participate, one-on-one cognitive interviews were conducted by a single qualified interviewer via the web-based platform, and participants were asked to describe their thought processes as they reviewed the questionnaire. The results indicated that the questionnaire instructions, questions, and response options were clear and easy to understand and answer. Select modifications were made based on participant feedback to enhance survey clarity and improve the accuracy of data collection.

### Data Collection

After finalization of the survey programming and user acceptance testing, HCPs were invited at their convenience to complete the self-administered, web-based questionnaire, which included eligibility screening questions and informed consent. To minimize the effect of subsequent questions on previous responses, participants were not able to go back and change their answers to questions they already answered. Data were collected between November 21, 2022, and December 6, 2022.

### Data Analysis

Data analyses were descriptive in nature and included summaries of the questionnaire responses presented in tabular displays of summary statistics. Questionnaire responses were summarized for the overall study population and stratified by HCP type (GIs, FM/IM/PCPs, PAs, NPs), gender (male and female), and years as a practicing HCP (≤10, 11-20, and >20). In addition, post hoc exploratory analyses of the response percentages were compared within the stratification variables using binomial regression models with the corresponding *P* values interpreted descriptively—rather than conferring an inferential assessment of a particular hypothesis—so there was no adjustment for multiple comparisons. Missing data were not imputed, and no survey weights were applied to the responses. All data analyses were performed using SAS 9.4 statistical software (SAS Institute Inc., Cary, NC; 2018). To ensure the integrity and quality of the analyses, data were reviewed for accuracy according to standard quality assurance procedures.

### Ethical Considerations

This study was reviewed by the RTI International Institutional Review Board (Federal-Wide Assurance #3331) and was determined to meet the criteria for exemption.

## Results

### HCP and Practice Characteristics

A total of 1194 HCPs (GIs, FM/IM/PCPs, NPs, and PAs treating patients with CD and UC) accessed the survey. Of these HCPs, 638 were ineligible, 96 were excluded because the target sample size was met for their provider type/specialty, and 1 refused consent, resulting in 459 HCPs who completed the survey. Of HCPs who completed the survey (*N* = 459), 55.3% were physicians (GIs, *n* = 100; FM/IM/PCPs, *n* = 154), 22.7% were NPs (*n* = 104), and 22.0% were PAs (*n* = 101), meeting all recruitment targets. Participating HCPs practiced in primary care/family medicine (49.5%), gastroenterology (37.7% including 36.6% of PAs and 34.6% of NPs), and internal medicine (12.9%). Additionally, most HCPs worked primarily in private practice (group, 49.7%; solo, 12.2%), while the remaining HCPs worked in inpatient hospital services (6.1%) and managed care or health maintenance organizations (2.6%). Participating HCP and practice characteristics are summarized in Table 1.

**Table 1.** HCP and practice characteristics.

Category	Overall (*N* = 459), *n* (%)
Type of HCP	
Physician (MD, DO)	254 (55.3)
NP	104 (22.7)
PA	101 (22.0)
Primary medical specialty^a^	
Gastroenterology	173 (37.7)
Internal medicine	59 (12.9)
Primary care/family care	227 (49.5)
Primary work environment	
Private practice, solo	56 (12.2)
Private practice, group	228 (49.7)
Managed care or health maintenance organization practice	12 (2.6)
Hospital, inpatient service	28 (6.1)
Outpatient clinic (eg, ambulatory care center, urgent care center)	78 (17.0)
Academic medical center	55 (12.0)
Other (eg, research)	2 (0.4)
Location (Census region)	
Midwest	100 (21.8)
Northeast	127 (27.7)
Southeast	101 (22.0)
Southwest	43 (9.4)
West	88 (19.2)
Age, years	
≤29	13 (2.8)
30-39	127 (27.7)
40-49	126 (27.5)
50-59	103 (22.4)
60-69	66 (14.4)
≥ 70	7 (1.5)
Prefer not to answer	17 (3.7)
Gender identity	
Female	215 (46.8)
Male	220 (47.9)
Gender fluid	2 (0.4)
Nonbinary	4 (0.9)
A gender identity not listed	0
I prefer not to answer	18 (3.9)
Race	
African American or Black	19 (4.1)
Alaska Native, American Indian, or Native American	6 (1.3)
Asian	77 (16.8)
Hispanic, Latin American, or Latinx	18 (3.9)
Middle Eastern or North African	7 (1.5)
Native Hawaiian or Pacific Islander	3 (0.7)
White	293 (63.8)
A race or ethnicity not listed	3 (0.7)
Prefer not to answer	47 (10.2)

Abbreviations: DO, doctor of osteopathy; FM, family medicine physician; GI, gastroenterologist; HCP, healthcare provider; IM, internal medicine physician; MD, medical doctor; NP, nurse practitioner; PA, physician assistant; PCP, primary care physician in family medicine or internal medicine.

^a^Participating HCPs practiced in gastroenterology (including 100% of GIs, 36.6% of PAs, and 34.6% of NPs), internal medicine (including 13.0% of FM/IM/PCPs, 19.8% of PAs, and 18.3% of NPs), and primary care/family medicine (including 87.0% of FM/IM/PCPs, 43.6% of PAs, and 47.1% of NPs).

Provider participants were asked how many adult patients (aged ≥18 years) they personally evaluate and/or treat in an average week; the mean (standard deviation [SD]) was 106.8 (65.19) patients with any medical condition, 12.2 (16.26) patients with CD, and 11.3 (16.22) patients with UC. Most participants (91.9%) reported spending >30 hours seeing patients each week. Table 2 summarizes the experiences of all participants and breaks down experiences by HCP type and specialty.

**Table 2.** Summary of HCP experiences.

	GI (*n* = 100)	FM/IM/PCP (*n* = 154)	NP (*n* = 104)	PA (*n* = 101)	Overall (*N* = 459)
No. of adult patients evaluated/treated in an average week					
Any medical condition					
Mean (SD)	105.1 (70.37)	117.3 (59.71)	109.3 (76.60)	89.9 (50.93)	106.8 (65.19)
Median	80	100	80	82.5	100
Crohn’s disease					
Mean (SD)	16.5 (22.29)	8.9 (10.45)	13.8 (15.40)	11.4 (16.41)	12.2 (16.26)
Median	10	5	5	10	8
Ulcerative colitis					
Mean (SD)	15.3 (20.96)	8.2 (12.94)	12.8 (15.04)	10.7 (15.72)	11.3 (16.22)
Median	10	5	7	5	6
No. of hours spent seeing patients each week, *n* (%)					
21-30	5 (5.0)	9 (5.8)	15 (14.4)	8 (7.9)	37 (8.1)
>30	95 (95.0)	145 (94.2)	89 (85.6)	93 (92.1)	422 (91.9)

Abbreviations: FM, family medicine physician; GI, gastroenterologist; HCP, healthcare provider; IM, internal medicine physician; NP, nurse practitioner; PA, physician assistant; PCP, primary care physician.

### Symptoms and Clinical Characteristics of IBD That Affect Treatment Choices

**Figure 1.**
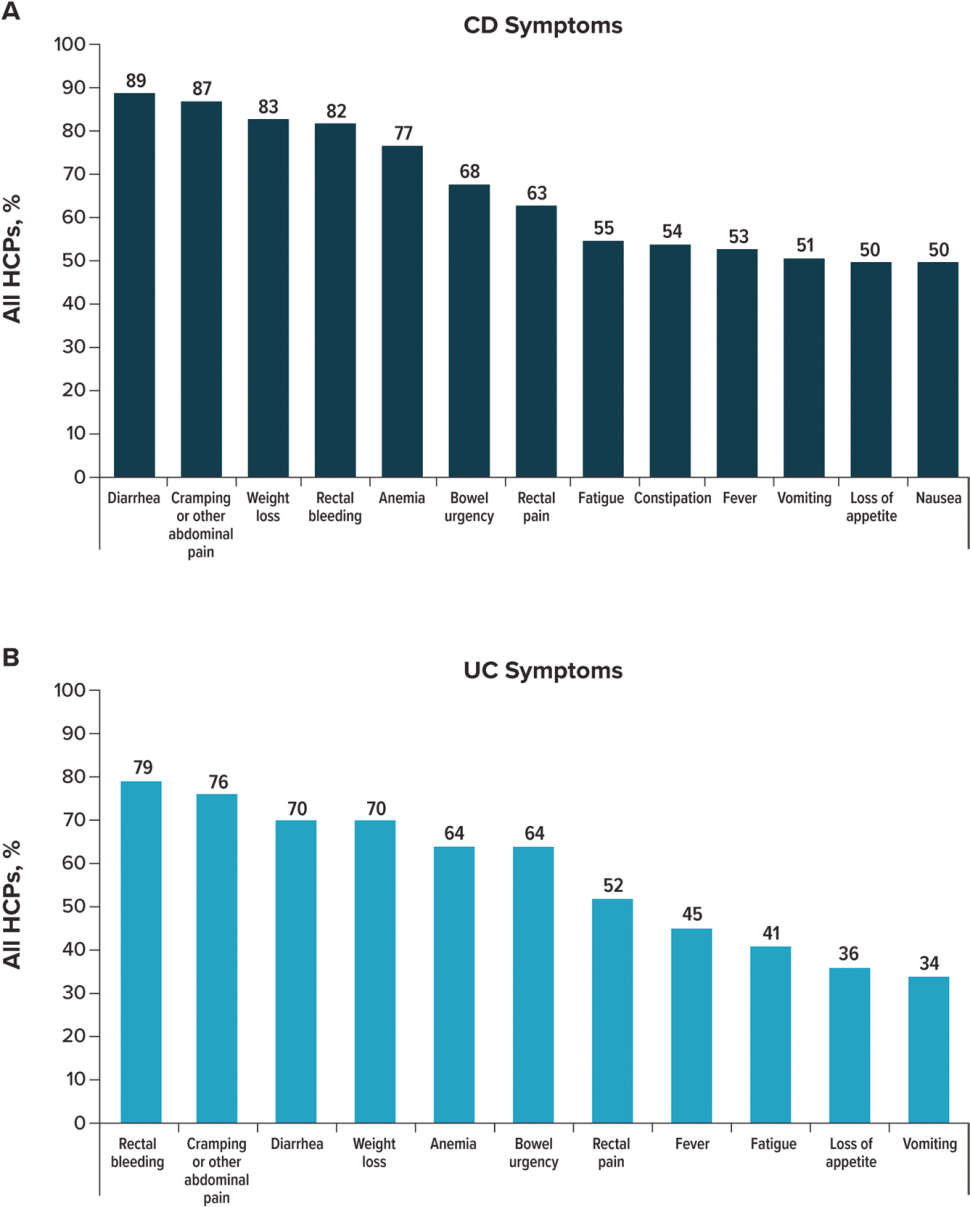
IBD symptoms are considered by overall HCPs when deciding on a patient’s course of treatment. For patients with (A) CD and (B) UC. HCP participants could select all that apply. “Other” and “none of the above” were selected by <4% of participants. Abbreviations: CD, Crohn’s disease; HCP, healthcare provider; IBD, inflammatory bowel disease; UC, ulcerative colitis.

**Figure 2.**
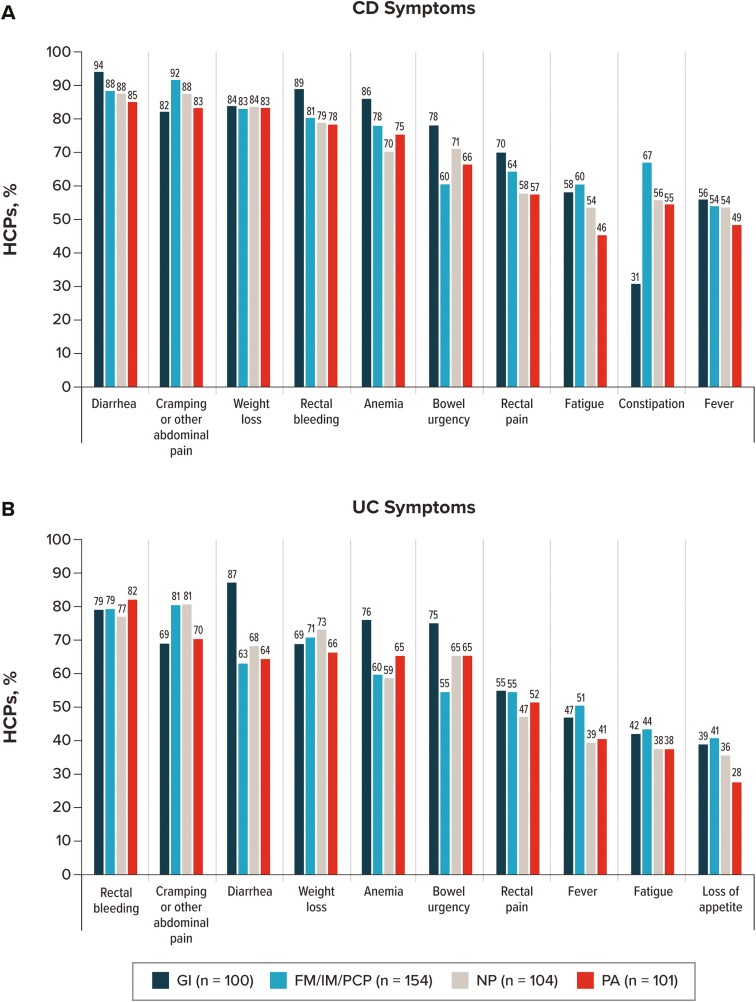
IBD symptoms are considered by each HCP type or specialty when deciding on a patient’s course of treatment. For patients with (A) CD and (B) UC. HCP participants could select all that apply. “Other” and “none of the above” were selected by <4% of participants. This figure shows the top 10 symptoms. Other CD symptoms included vomiting, loss of appetite, and nausea; other UC symptoms included vomiting. Abbreviations: CD, Crohn’s disease; FM, family medicine physician; GI, gastroenterologist; HCP, healthcare provider; IBD, inflammatory bowel disease; IM, internal medicine physician; NP, nurse practitioner; PA, physician assistant; PCP, primary care physician; UC, ulcerative colitis.

Participants were asked which symptoms of CD and UC from a provided list they most frequently considered when deciding on a course of treatment (Figure 1). When considering patients with CD, HCPs most frequently selected diarrhea (88.7%), followed by cramping or other abdominal pain (86.7%), weight loss (83.4%), rectal bleeding (81.5%), and anemia (77.3%; Figure 1A). A lower percentage of GIs (31.0%) than FM/IM/PCPs (66.9%, *P* < .0001), NPs (55.8%, *P* = .0003), or PAs (54.5%, *P* = .0006) selected constipation; on the other hand, symptoms considered by a higher percentage of GIs than by at least one other HCP type/specialty included diarrhea, rectal bleeding, anemia, and bowel urgency (Figure 2A).

When considering patients with UC, HCPs most frequently selected rectal bleeding (79.3%), followed by cramping or other abdominal pain (75.8%), weight loss (69.9%), diarrhea (69.7%), and anemia (64.3%; Figure 1B). A higher percentage of GIs (87.0%) than FM/IM/PCPs (63.0%, *P* < .0001), NPs (68.3%, *P* = .0010), or PAs (64.4%, *P* = .0001) selected diarrhea (Figure 2B). Other symptoms considered by a higher percentage of GIs than by at least one other HCP type/specialty included anemia, bowel urgency, and nausea.

**Figure 3.**
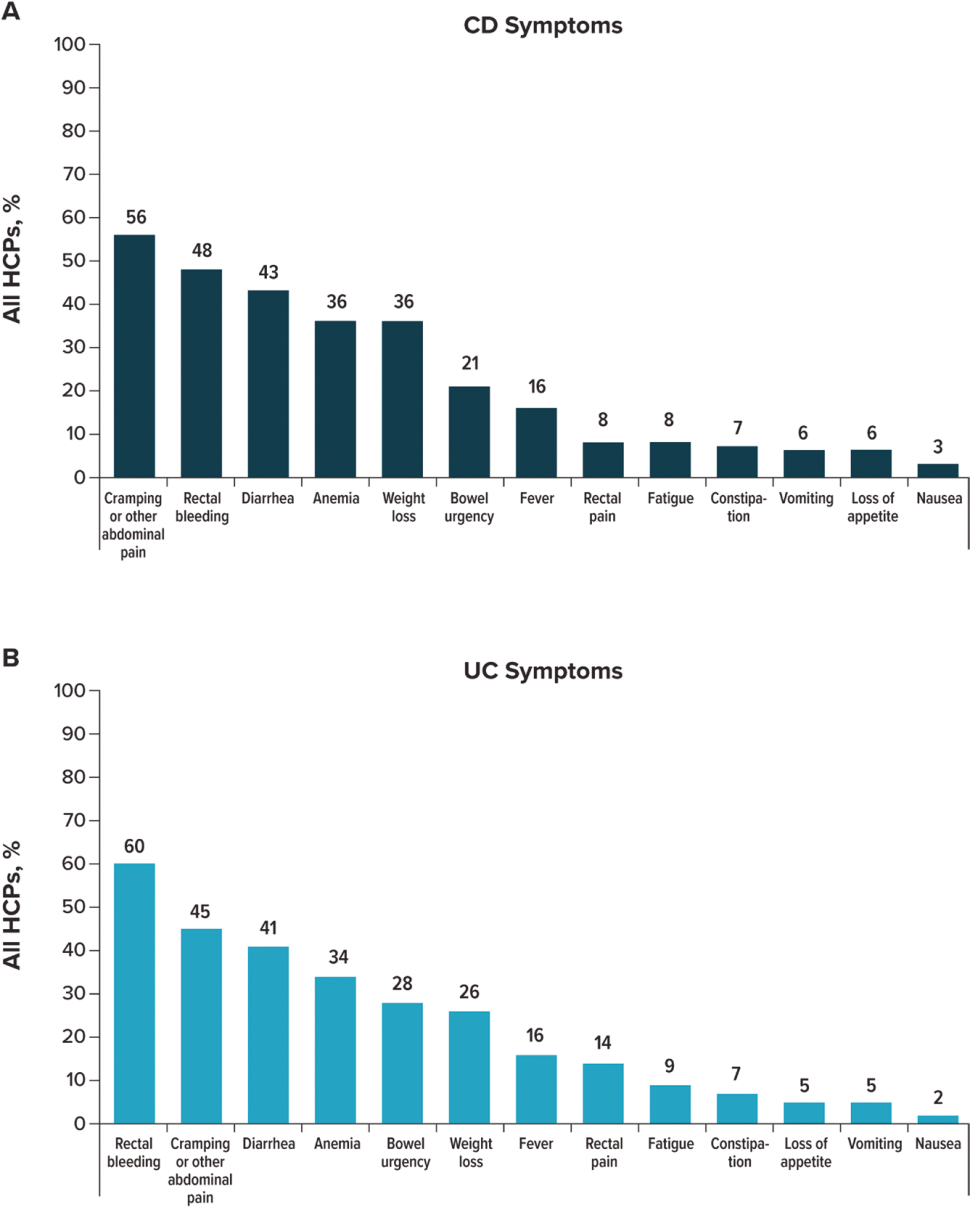
IBD symptoms are considered most important by overall HCPs when deciding on a patient’s course of treatment. For patients with (A) CD and (B) UC. HCP participants could select up to 3 symptoms. Abbreviations: CD, Crohn’s disease; HCP, healthcare provider; IBD, inflammatory bowel disease; UC, ulcerative colitis.

Using the same list of IBD symptoms, excluding the option “other,” participants were asked to select up to 3 symptoms of CD and UC they considered “most” important when deciding on a course of treatment (Figure 3). For patients with CD, the top 5 “most” important symptoms selected by HCPs were cramping or other abdominal pain (56.2%), rectal bleeding (48.1%), diarrhea (42.9%), anemia (35.7%), and weight loss (35.7%). For patients with UC, HCPs selected rectal bleeding (60.0%), cramping or other abdominal pain (45.1%), diarrhea (40.5%), anemia (33.9%), and bowel urgency (28.4%). For both patients with CD and UC, a higher percentage of GIs (CD: 58.0%; UC: 59.6%) than FM/IM/PCPs (CD: 36.4%, *P = *.0006; UC: 32.5%, *P* < .0001), NPs (CD: 38.5%, *P = *.0046; UC: 42.3%, *P* = .0127), or PAs (CD: 42.6%, *P = *.0274; UC: 32.0%, *P* < .0001) selected diarrhea as the “most” important symptom; for patients with UC, a higher percentage of GIs (41.4%) than FM/IM/PCPs (20.1%, *P = *.0004) or NPs (27.9%, *P* = .0416) selected bowel urgency as “most” important (Supplementary Figure S1).

**Figure 4.**
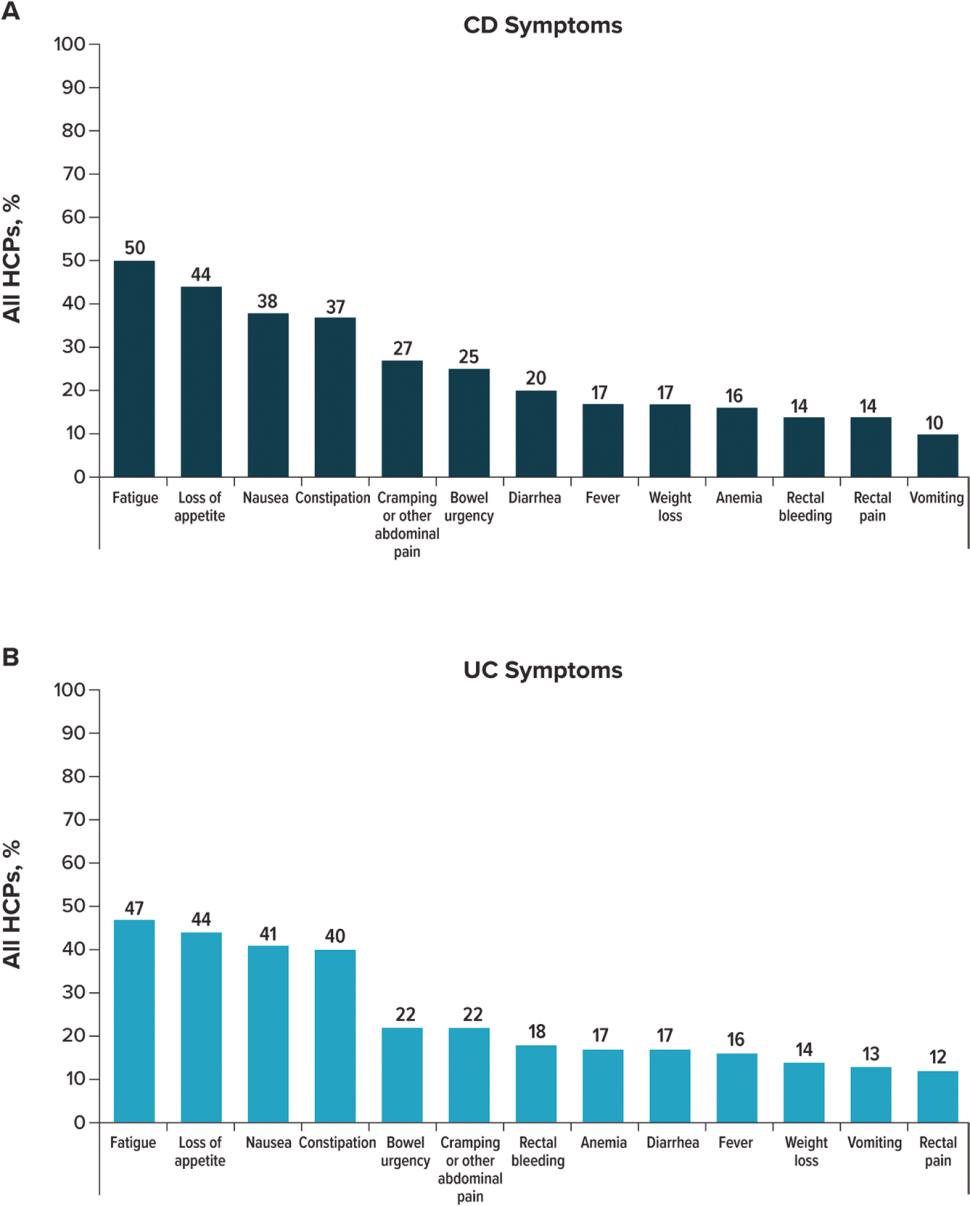
IBD symptoms are considered least important by overall HCPs when deciding on a patient’s course of treatment. For patients with (A) CD and (B) UC. HCP participants could select up to 3 symptoms. Abbreviations: CD, Crohn’s disease; HCP, healthcare provider; IBD, inflammatory bowel disease; UC, ulcerative colitis.

After symptoms selected as “most” important were removed, participants were then asked to identify up to 3 symptoms of CD and UC they considered “least” important when deciding on a course of treatment (Figure 4). For patients with CD, the 5 “least” important symptoms selected by HCPs were fatigue (50.0%), loss of appetite (43.8%), nausea (38.2%), constipation (37.2%), and cramping or other abdominal pain (27.4%). For patients with UC, HCPs selected fatigue (46.8%), loss of appetite (44.3%), nausea (40.6%), constipation (40.0%), and bowel urgency (22.0%).

**Figure 5.**
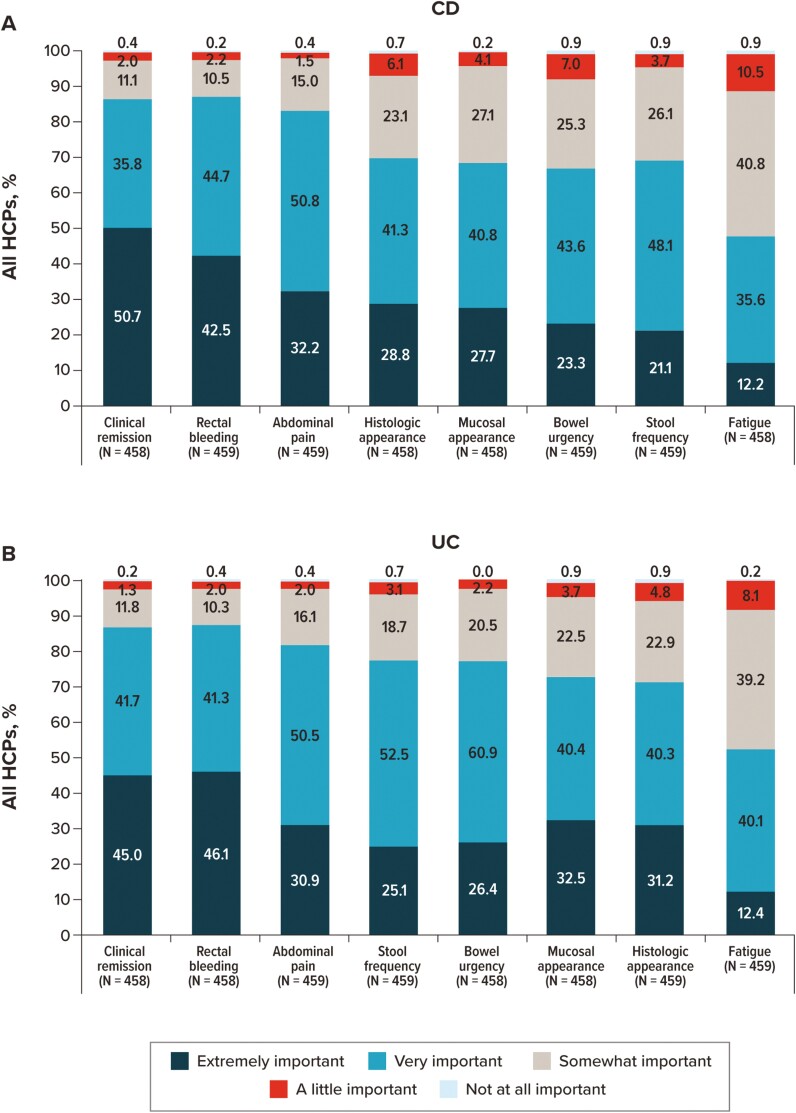
Importance of IBD symptoms and clinical characteristics considered by overall HCPs when deciding on a patient’s course of treatment. For patients with (A) CD and (B) UC. Abbreviations: CD, Crohn’s disease; HCP, healthcare provider; IBD, inflammatory bowel disease; UC, ulcerative colitis.

Participants were asked to independently rate the importance of listed items as they relate to deciding on the course of treatment for CD and UC (Figure 5). Except for fatigue, all items were rated as “very” or “extremely” important by more than two-thirds of participants (rectal bleeding [CD: 87.1%; UC: 87.3%], clinical remission [CD: 86.5%; UC: 86.7%], abdominal pain [CD: 83.0%; UC: 81.5%], histologic appearance [CD: 70.1%; UC: 71.5%], stool frequency [CD: 69.3%; UC: 77.6%], mucosal appearance [CD: 68.5%; UC: 72.9%], and bowel urgency [CD: 66.9%; UC: 77.3%]). For patients with CD, a higher percentage of GIs rated mucosal appearance, stool frequency, and clinical remission as “very” or “extremely” important than did FM/IM/PCPs, NPs, or PAs. For patients with UC, on the other hand, GIs, NPs, and PAs more frequently rated bowel urgency, mucosal appearance, and stool frequency as “very” or “extremely” important than did FM/IM/PCPs (Supplementary Table S1).

Participants were asked to rate the level of importance for treatment decision-making of a patient achieving complete disease control (defined as mucosal/histologic healing, clinical remission, and no bowel urgency; Supplementary Figure S2). Most HCPs responded that a patient achieving complete control was “very” important (CD: 47%; UC: 46.0%) or “extremely” important (CD: 40%; UC: 39.4%) for their decision on treatment for CD and UC; the importance was rated higher among GIs (95.0%, *P* = .0001), NPs (90.4%, *P* = .0157), and PAs (89.1%, *P = *.0396) than among FM/IM/PCPs (79.9%) for patients with CD and higher among GIs (94.0%) than among FM/IM/PCPs (79.2%, *P* = .0003) and PAs (84.2%, *P* = .0238) for patients with UC.

### Reasons for Dose Escalation or Switching Treatments

#### Reasons for dose escalation

**Figure 6.**
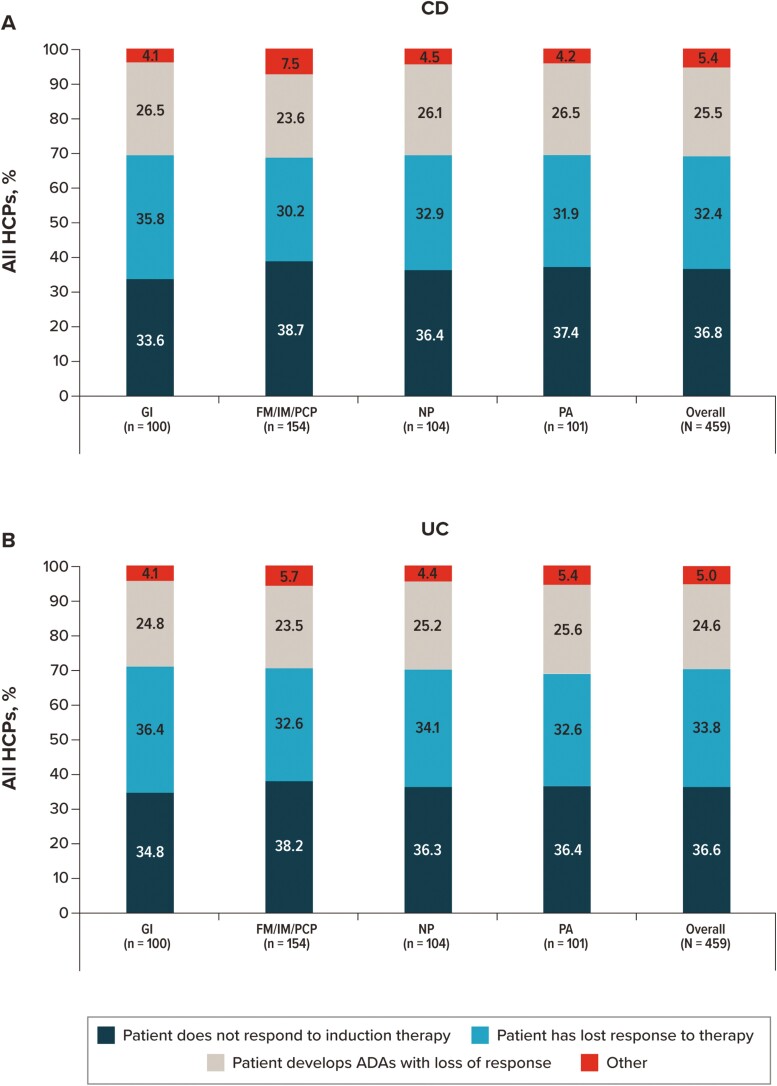
Reasons considered by each HCP type or specialty when escalating the dose of a patient’s current treatment. For patients with (A) CD and (B) UC. Abbreviations: ADA, antidrug antibody; CD, Crohn’s Disease; FM, family medicine physician; GI, gastroenterologist; HCP, healthcare provider; IM, internal medicine physician; NP, nurse practitioner; PA, physician assistant; PCP, primary care physician; UC, ulcerative colitis.

Participants were presented with a list of 4 reasons for escalating the dose of a patient’s current treatment for CD and UC and asked to estimate the percentage of how much each reason would factor into their decision-making, with percentages summing to 100% (Figure 6). Reasons to escalate the dose of a patient’s current treatment that factored most heavily into HCPs’ decisions were no response to induction therapy (mean, SD; CD: 36.8%, 17.62%; UC: 36.6%, 17.55%) and loss of response to therapy (CD: 32.4%, 14.16%; UC: 33.8%, 15.11%). The development of antidrug antibodies with loss of response was also considered in the decision to escalate the treatment dose, although to a lesser extent (mean, SD; CD: 25.5%, 15.91%; UC: 24.6%, 15.55%).

**Figure 7.**
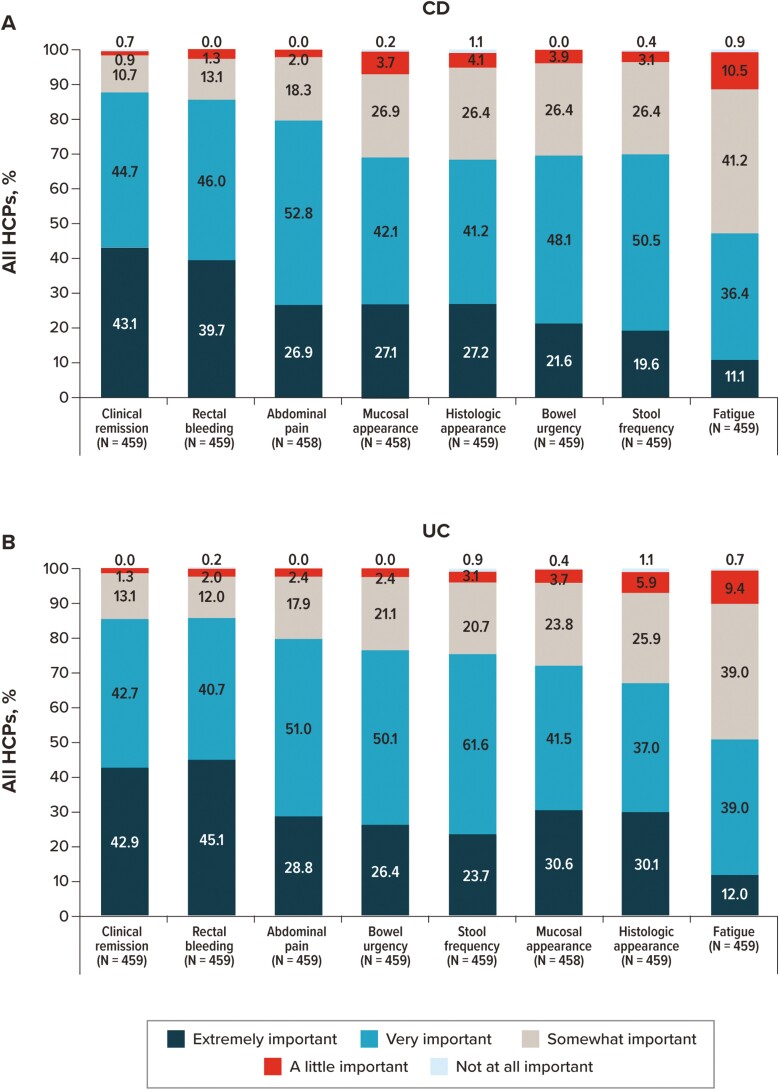
Importance of IBD symptoms and clinical characteristics considered by overall HCPs when escalating the dose of a patient’s current treatment. For patients with (A) CD and (B) UC. Abbreviations: CD, Crohn’s disease; HCP, healthcare provider; IBD, inflammatory bowel disease; UC, ulcerative colitis.

Participants were then asked to independently rate the importance of several items as they relate to escalating the dose of current treatment for CD and UC (Figure 7). Except for fatigue, all items were rated as “very” or “extremely” important by more than two-thirds of participants (rectal bleeding [CD: 85.7%; UC: 85.8%], clinical remission [CD: 87.8%; UC: 85.6%], abdominal pain [CD: 79.7%; UC: 79.7%], histologic appearance [CD: 68.4%; UC: 67.1%], stool frequency [CD: 70.1%; UC: 75.4%], mucosal appearance [CD: 69.2%; UC: 72.1%], and bowel urgency [CD: 69.7%; UC: 76.5%]). Participants also rated the level of importance of a patient not achieving complete disease control for their decision to escalate the dose of current treatment for patients with CD and UC (Supplementary Figure S3). Most HCPs responded that a patient not achieving complete disease control was “very” important (CD: 50.7%; UC: 49.0%) or “extremely” important (CD: 31.2%; UC: 34.9%) for their decision.

#### Reasons for switching treatments

**Figure 8.**
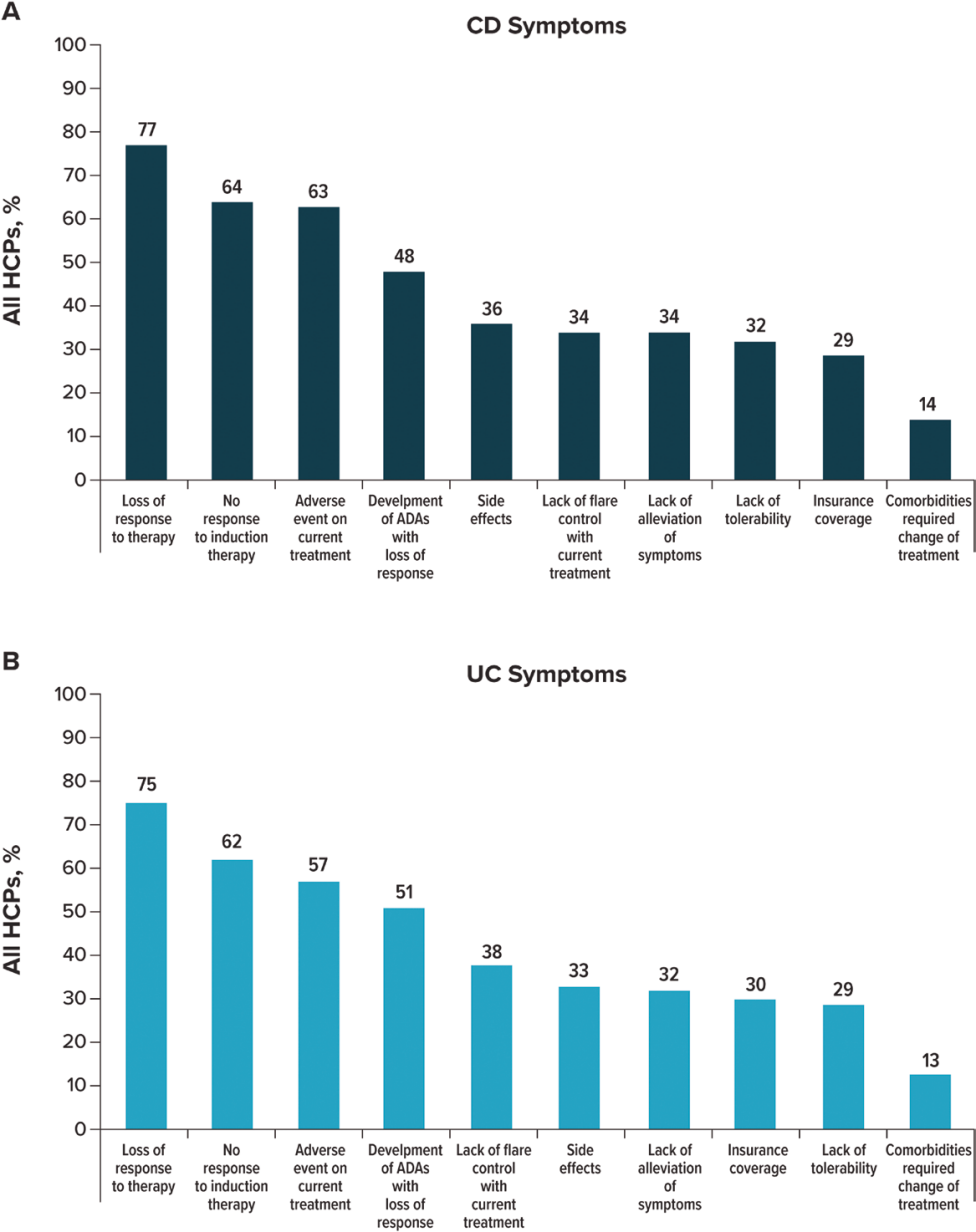
Reasons considered by overall HCPs when switching a patient’s current treatment. For patients with (A) CD and (B) UC. HCP participants could select up to 5 options. “Other treatment is more cost-effective for patient,” “mode of action and administration,” “patient requests to switch treatment,” “frequency of injections of current treatment,” “fewer administrative hurdles with other treatment,” “other,” and “none of the above” were selected by <10% of participants. Abbreviations: ADA, antidrug antibody; CD, Crohn’s Disease; HCP, healthcare provider; UC, ulcerative colitis.

To determine the reasons that HCPs would switch a patient’s current treatment to a different advanced treatment for CD or UC (Figure 8), participants were asked to indicate the most important reasons for such a switch from a list that included 10 options. For both CD and UC, the primary reasons that HCPs switch patients to a different advanced therapy were loss of response to therapy (CD: 77%; UC: 74.5%), no response to induction therapy (CD: 64%; UC: 61.9%), and adverse event on current treatment (CD: 63%; UC: 57.3%).

**Figure 9.**
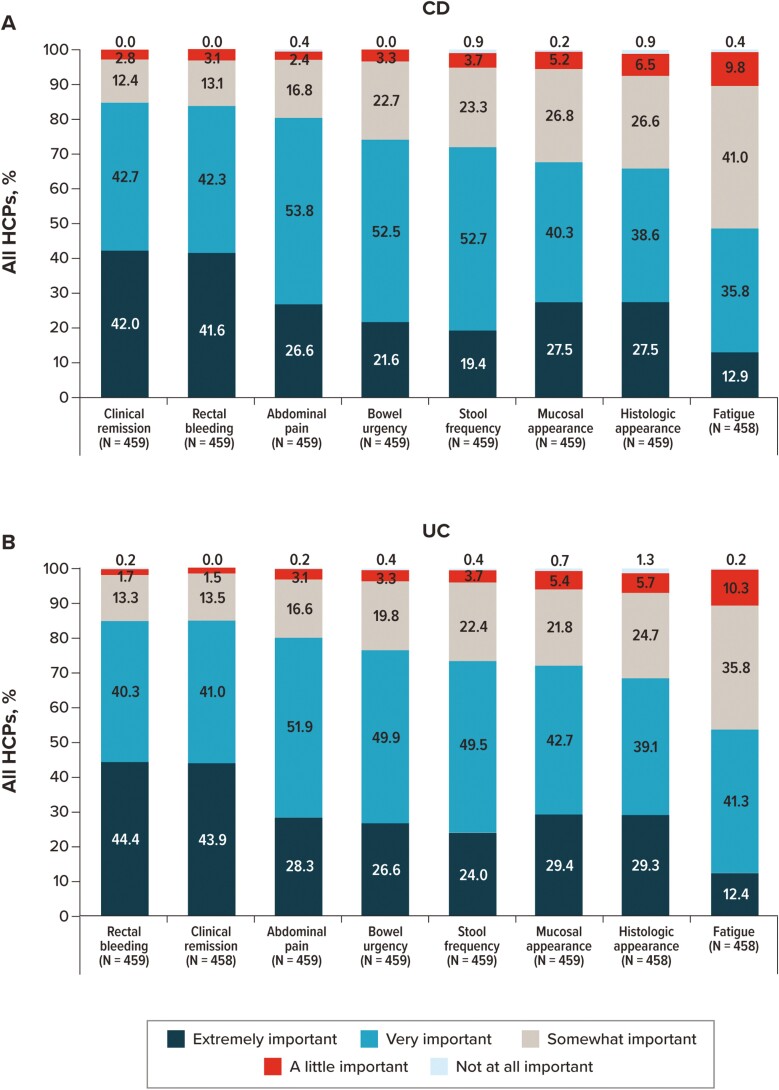
Importance of IBD symptoms and clinical characteristics considered by overall HCPs when switching a patient’s current treatment. For patients with (A) CD and (B) UC. Abbreviations: CD, Crohn’s disease; HCP, healthcare provider; IBD, inflammatory bowel disease; UC, ulcerative colitis.

Using the same list, participants were then asked to independently rate the importance of items as they relate to switching a patient’s current treatment for CD and UC (Figure 9). Except for fatigue, all items were rated as “very” or “extremely” important by more than two-thirds of participants (rectal bleeding [CD: 83.9%; UC: 84.7%], clinical remission [CD: 84.7%; UC: 84.9%], abdominal pain [CD: 80.4%; UC: 80.2%], histologic appearance [CD: 66.1%; UC: 68.3%], stool frequency [CD: 72.1%; UC: 73.4%], mucosal appearance [CD: 67.8%; UC: 72.1%], and bowel urgency [CD: 74.1%; UC: 76.5%]). Finally, participants rated the level of importance of a patient not achieving complete disease control for their decision to switch to a current treatment for patients with CD and UC (Supplementary Figure S4). Most HCPs responded that a patient not achieving complete disease control was “very” important (CD: 52.2%; UC: 49.9%) or “extremely” important (CD: 29.3%; UC: 33.3%) for their decision.

### Urgency NRS

**Figure 10.**
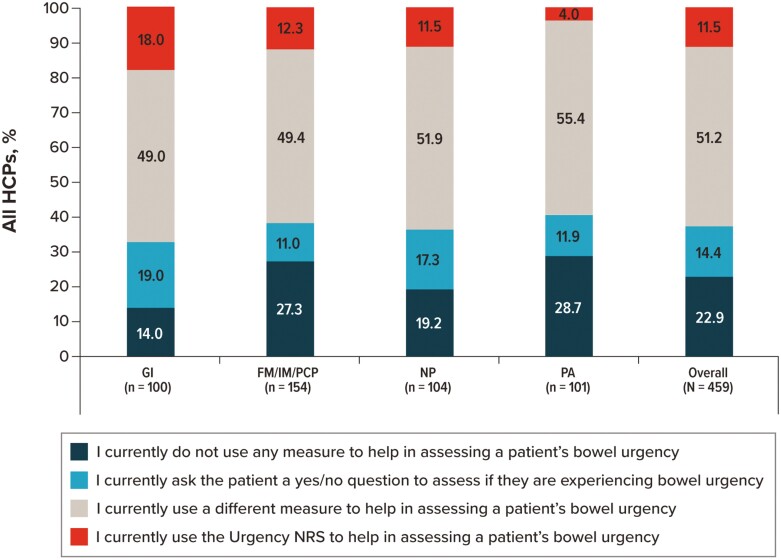
HCPs’ current use of measures to assess bowel urgency for their patients with IBD. Abbreviations: FM, family medicine physician; GI, gastroenterologist; HCP, healthcare provider; IBD, inflammatory bowel disease; IM, internal medicine physician; NP, nurse practitioner; PA, physician assistant; PCP, primary care physician.

When asked about their current use of measures to assess patient bowel urgency, 51.2% of participants indicated that they currently ask patients a yes/no question to assess bowel urgency, 22.9% indicated that they currently use the Urgency NRS, 14.4% indicated that they use a different measure to assess bowel urgency, and 11.5% indicated that they do not use any measure to assess patient bowel urgency (Figure 10). Current use of the Urgency NRS was higher among PAs (28.7%) and FM/IM/PCPs (27.3%) than NPs (19.2%) and GIs (14.0%). Among participants who indicated that they use a measure other than the Urgency NRS, the UC Patient-Reported Outcomes Signs and Symptoms (UC-PRO/SS) measure was the most frequently selected response (45.5%), followed by the Symptoms and Impacts Questionnaire for CD or UC (SIQ-CD or SIQ-UC; 39.4%) and the Simple Clinical Colitis Activity Index (SCCAI; 25.8%; (Supplementary Table S2).

When participants who were not currently using the Urgency NRS were asked how willing they would be to use it in the future, over half of participants indicated that they were “very willing” (42.9%) or “extremely willing” (11.6%); 34.7% indicated that they were “somewhat willing”; and few indicated they were “a little willing” (8.8%) or “not at all willing” (2.0%; Supplementary Figure S5). Among participants who indicated that they were “a little willing” or “not at all willing” to use the Urgency NRS, the following reasons were selected: “I do not think this measure would be helpful to me” (42.1%), “I am not familiar with this measure” (39.5%), “I do not want to add another task to complete during the patient’s appointment” (23.7%), and “I do not have enough time during an office visit to use this measure with my patients” (21.1%; Supplementary Table S3).

## Discussion

The present real-world study characterized the symptoms and clinical characteristics that factor into HCPs’ treatment choices for their patients with CD and UC. Most HCPs considered diarrhea, cramping or abdominal pain, weight loss, rectal bleeding, anemia, and bowel urgency as key symptoms when choosing a course of treatment for these patient populations. Cramping or abdominal pain was considered one of the most important symptoms of CD, whereas rectal bleeding was ranked one of the most important symptoms of UC.

Most participants ranked rectal bleeding, clinical remission, and abdominal pain as “very” to “extremely” important in decisions regarding the course of treatment, dose escalation, and switching treatments, for both CD and UC. For both CD and UC, the primary factors considered in the decision to escalate the dose of a patient’s current treatment were loss of response to therapy and a lack of response to induction therapy, while the most important reasons for switching a patient’s current treatment to a different advanced therapy were loss of response to therapy, no response to induction therapy, and an adverse event on the patient’s current treatment. Our results align with HCP-specific findings from a recent international survey by Rubin et al.,^20^ in which physicians (*n* = 654; 37%)—as well as patients with CD (*n* = 1368; 37%) and patients with UC (*n* = 1030; 36%)—reported that achieving a durable response was an important driver of treatment choice.

Similarly, most participants in our study reported that a patient achieving complete disease control is a “very” or “extremely” important factor in treatment decision-making. Furthermore, a lack of complete disease control was considered “very” or “extremely” important in the decision to escalate treatment dose or switch treatments. A survey of IBD patients found that the highest-priority outcome was complete control of symptoms,^21^ which underscores the importance of our findings.

When deciding on a course of treatment for patients with CD and UC, a higher percentage of GIs considered diarrhea “most” important than did FM/IM/PCPs, NPs, or PAs. The mucosal appearance was rated as “very” to “extremely” important by a higher percentage of GIs than by other HCP types/specialties; this finding aligns with the fact that GIs are specially trained to perform endoscopic examination of the gastrointestinal tract.^22^ Also, achieving complete disease control was considered more important in treatment decision-making by GIs than by the other HCP types/specialties. Overall, our findings suggest that there is an opportunity for education among other specialties (particularly FM/IM/PCPs) about the clinical presentation of CD and UC as well as about the symptoms of most importance when determining how to treat and manage patients with each disease. These findings are clinically relevant, as IBD treatment often involves various HCP types and specialties,^17^ and differences in HCP treatment choices can result in suboptimal IBD care.^23^

It is important to note that HCPs and their patients with IBD may have divergent opinions regarding which symptoms are most important in guiding treatment decisions.^20,24,25^ For example, patients with IBD often report fatigue as an important symptom that impacts their quality of life.^20,26,27^ However, HCPs may not recognize the burden of fatigue,^20^ which could partially account for why HCPs in our study considered fatigue of little importance in treatment choices. Additionally, some patients may be embarrassed to discuss important symptoms, like bowel urgency, with their HCP, which could lead to a gap in communication.^24,25^ Although not evaluated in this study, differences in preferences and communication between HCPs and patients may impact the symptoms that are considered most important in treatment decision-making.^20,24,25^

Bowel urgency is a prominent symptom of CD and UC, and addressing it is important for improving patient quality of life.^26,27^ Indeed, Rubin et al.^20^ found that 65% of patients with CD and 72% of patients with UC reported rectal urgency as the symptom with the greatest impact on their quality of life. While a portion of HCPs in our study considered bowel urgency as “least” important (22.0%) when deciding on a course of treatment for patients with UC, over a quarter of HCPs found bowel urgency to be “most” important (28.4%). Furthermore, more than two-thirds of HCPs rated bowel urgency as “very” or “extremely” important in decisions regarding the course of treatment (CD: 66.9%; UC: 77.3%), dose escalation (CD: 69.7%; UC: 76.5%), and switching treatments (CD: 74.1%; UC: 76.5%) for patients with CD and UC. Bowel urgency was also rated as “very” or “extremely” important by a higher percentage of GIs, NPs, and PAs than by FM/IM/PCPs when deciding the course of treatment for UC.

The US Food and Drug Administration recommends using fit-for-purpose patient-reported outcome instruments to evaluate symptoms that are important to patients with CD and UC (eg, bowel urgency), particularly when assessing treatment benefits in clinical trials.^12,13^ While more than half of participants in our study stated that they currently use a yes/no question to assess bowel urgency, nearly a quarter of participants indicated that they currently use the Urgency NRS, a valid and reliable patient-reported outcome measure to assess the severity of bowel urgency in CD and UC.^15,16^ Among participants not currently using the Urgency NRS, 89.3% were at least somewhat willing to use it in the future. Although a lower percentage of FM/IM/PCPs responded that they consider bowel urgency in their treatment decision-making compared with GIs, their use of the Urgency NRS was among the highest.

This study was designed to select a diverse and generally representative sample of HCPs; however, there is no exhaustive list of all HCPs who treat patients with CD or UC. Therefore, the participants in the present study may not be representative of all such HCPs in the United States. Furthermore, our study sample included a smaller percentage of HCPs who practiced in solo private practice, managed care or health maintenance organizations, or inpatient hospital settings; therefore, HCPs working in these environments may have been underrepresented. In addition, participants were limited to those who were willing to participate and agreed to take an online survey. This could result in selection bias, meaning that the opinions of these participants may differ from those who do not agree to participate in panels and online surveys. In addition, this study did not distinguish HCP treatment choices based on patient disease severity (eg, mild, moderate, or severe CD or UC), and future research could explore how the level of disease severity may impact clinical factors of importance for treatment decision-making. Lastly, study results are based on self-report, which is susceptible to responder bias that may impact the accuracy of the results. However, there is a paucity of research regarding the factors that impact HCPs’ treatment choices for their patients with CD and UC, and the present study, which included a large sample of HCPs, provides an important contribution to the literature on this topic.

## Conclusion

This observational survey among HCPs provides valuable real-world insight into the factors impacting treatment choices for patients with CD and UC. Symptoms identified by HCPs that were of most importance when deciding on the course of treatment for their patients with CD and UC included diarrhea, cramping or abdominal pain, weight loss, rectal bleeding, anemia, and bowel urgency. Furthermore, most participants ranked rectal bleeding, clinical remission, abdominal pain, and complete control as “very” to “extremely” important in decisions regarding the course of treatment, dose escalation, and switching treatments. Lastly, almost a quarter of HCPs indicated that they currently use the Urgency NRS, while most others were at least somewhat willing to use it in the future to assess the severity of bowel urgency for their patients with CD and UC. Understanding IBD symptoms and clinical characteristics can help provide tailored insights to HCPs across different types and specialties, which may ultimately improve treatment approaches and outcomes for patients with CD and UC.

## Supplementary Material

otae053_suppl_Supplementary_Material

## Data Availability

Data are not publicly available.
