# Supplementary material for: Evaluation of the Symptoms and Clinical Characteristics of Crohn’s Disease and Ulcerative Colitis That Affect Healthcare Providers’ Treatment Choices
Source: Crohns Colitis 360. 2024 Oct 5;6(4):otae053. doi: 10.1093/crocol/otae053 (PMC11471961; doi:10.1093/crocol/otae053)
Supplement: otae053_suppl_Supplementary_Material [file otae053_suppl_supplementary_material.docx]

# Supplementary Data Content

## Table S-1. Importance of Key IBD Symptoms and Clinical Characteristics When Deciding on a Patient’s Course of Treatment by Each HCP Type or Specialty

|  | GI | FM/IM/PCP | PA | NP | Overall |
| --- | --- | --- | --- | --- | --- |
| CD, n (%) | | | | | |
| Mucosal appearance^a^ | n = 99 | n = 154 | n = 101 | n = 104 | N = 458 |
| Extremely important | 51 (51.5) | 24 (15.6) | 25 (24.8) | 27 (26.0) | 127 (27.7) |
| Very important | 36 (36.4) | 62 (40.3) | 42 (41.6) | 47 (45.2) | 187 (40.8) |
| Somewhat important | 11 (11.1) | 58 (37.7) | 29 (28.7) | 26 (25.0) | 124 (27.1) |
| A little important | 1 (1.0) | 10 (6.5) | 4 (4.0) | 4 (3.8) | 19 (4.1) |
| Not at all important | 0 | 0 | 1 (1.0) | 0 | 1 (0.2) |
| Stool frequency^b^ | n = 100 | n = 154 | n = 101 | n = 104 | N = 459 |
| Extremely important | 28 (28.0) | 28 (18.2) | 12 (11.9) | 29 (27.9) | 97 (21.1) |
| Very important | 56 (56.0) | 68 (44.2) | 55 (54.5) | 42 (40.4) | 221 (48.1) |
| Somewhat important | 14 (14.0) | 46 (29.9) | 28 (27.7) | 32 (30.8) | 120 (26.1) |
| A little important | 1 (1.0) | 9 (5.8) | 6 (5.9) | 1 (1.0) | 17 (3.7) |
| Not at all important | 1 (1.0) | 3 (1.9) | 0 | 0 | 4 (0.9) |
| Clinical remission^c^ | n = 100 | n = 154 | n = 100 | n = 104 | N = 458 |
| Extremely important | 75 (75.0) | 62 (40.3) | 47 (47.0) | 48 (46.2) | 232 (50.7) |
| Very important | 19 (19.0) | 66 (42.9) | 38 (38.0) | 41 (39.4) | 164 (35.8) |
| Somewhat important | 4 (4.0) | 21 (13.6) | 13 (13.0) | 13 (12.5) | 51 (11.1) |
| A little important | 1 (1.0) | 4 (2.6) | 2 (2.0) | 2 (1.9) | 9 (2.0) |
| Not at all important | 1 (1.0) | 1 (0.6) | 0 | 0 | 2 (0.4) |
| UC, n (%) | | | | | |
| Bowel urgency^d^ | n = 100 | n = 153 | n = 101 | n = 104 | N = 458 |
| Extremely important | 40 (40.0) | 33 (21.6) | 23 (22.8) | 25 (24.0) | 121 (26.4) |
| Very important | 52 (52.0) | 68 (44.4) | 57 (56.4) | 56 (53.8) | 233 (50.9) |
| Somewhat important | 6 (6.0) | 45 (29.4) | 21 (20.8) | 22 (21.2) | 94 (20.5) |
| A little important | 2 (2.0) | 7 (4.6) | 0 | 1 (1.0) | 10 (2.2) |
| Not at all important | 0 | 0 | 0 | 0 | 0 |
| Mucosal appearance^a^ | n = 100 | n = 153 | n = 101 | n = 104 | N = 458 |
| Extremely important | 54 (54.0) | 29 (19.0) | 30 (29.7) | 36 (34.6) | 149 (32.5) |
| Very important | 34 (34.0) | 61 (39.9) | 45 (44.6) | 45 (43.3) | 185 (40.4) |
| Somewhat important | 11 (11.0) | 51 (33.3) | 21 (20.8) | 20 (19.2) | 103 (22.5) |
| A little important | 1 (1.0) | 10 (6.5) | 4 (4.0) | 2 (1.9) | 17 (3.7) |
| Not at all important | 0 | 2 (1.3) | 1 (1.0) | 1 (1.0) | 4 (0.9) |
| Stool frequency^b^ | n = 100 | n = 154 | n = 101 | n = 104 | N = 459 |
| Extremely important | 43 (43.0) | 25 (16.2) | 23 (22.8) | 24 (23.1) | 115 (25.1) |
| Very important | 42 (42.0) | 80 (51.9) | 58 (57.4) | 61 (58.7) | 241 (52.5) |
| Somewhat important | 15 (15.0) | 38 (24.7) | 15 (14.9) | 18 (17.3) | 86 (18.7) |
| A little important | 0 | 9 (5.8) | 4 (4.0) | 1 (1.0) | 14 (3.1) |
| Not at all important | 0 | 2 (1.3) | 1 (1.0) | 0 | 3 (0.7) |

CD = Crohn’s disease; FM = family medicine physician; GI = gastroenterologist; HCP = healthcare provider; IBD = inflammatory bowel disease; IM = internal medicine physician; NP = nurse practitioner; PA = physician assistant; PCP = primary care physician; UC = ulcerative colitis.

^a^ For CD, more GIs rated mucosal appearance as “very” or “extremely” important than did FM/IM/PCPs (*P*<0.0001), NPs (*P*=0.0026), or PAs (*P*=0.0002). For UC, more GIs (*P*<0.0001), NPs (*P*=0.0009), and PAs (*P*=0.0091) rated mucosal appearance as “very” or “extremely” important than did FM/IM/PCPs. Also for UC, more GIs (*P*=0.0117) rated mucosal appearance as “very” or “extremely” important than did PAs.

^b^ For CD, more GIs rated stool frequency as “very” or “extremely” important than did FM/IM/PCPs (*P*<0.0001), NPs (*P=*0.0075), or PAs (*P*=0.0032). For UC, more GIs (*P*=0.0013), NPs (*P*=0.0114), and PAs (*P*=0.0283) rated stool frequency as “very” or “extremely” important than did FM/IM/PCPs.

^c^ For CD, more GIs rated clinical remission as “very” or “extremely” important than did FM/IM/PCPs (*P*=0.0048), NPs (*P*=0.0447), or PAs (*P*=0.0364).

^d^ For UC, more GIs (*P*<0.0001), NPs (*P*=0.0342), and PAs (*P=*0.0182) rated bowel urgency as “very” or “extremely” important than did FM/IM/PCPs. Also, more GIs rated bowel urgency as “very” or “extremely” important than did NPs (*P*=0.0041) and PAs (*P*=0.0088).

## Table S-2. Use of Different Measures to Assess Patient Bowel Urgency by HCP Type or Specialty

|  | GI  **(n = 100)** | FM/IM/ PCP  **(n = 154)** | PA  **(n = 101)** | NP  **(n = 104)** | Overall  **(N = 459)** |
| --- | --- | --- | --- | --- | --- |
| n (%) |  |  |  |  |  |
| Simple Clinical Colitis Activity Index (SCCAI) | 6 (31.6) | 5 (29.4) | 2 (16.7) | 4 (22.2) | 17 (25.8) |
| Ulcerative Colitis Patient-Reported Outcomes Signs and Symptoms (UC-PRO/SS) | 8 (42.1) | 7 (41.2) | 7 (58.3) | 8 (44.4) | 30 (45.5) |
| Symptoms and Impacts Questionnaire for Crohn's Disease or Ulcerative Colitis (SIQ-CD or SIQ-UC) | 5 (26.3) | 9 (52.9) | 5 (41.7) | 7 (38.9) | 26 (39.4) |
| Other | 1 (5.3) | 1 (5.9) | 1 (8.3) | 1 (5.6) | 4 (6.1) |

FM = family medicine physician; GI = gastroenterologist; HCP = healthcare provider; IM = internal medicine physician; NP = nurse practitioner; PA = physician assistant; PCP = primary care physician.

## Table S-3. Reasons for Low Willingness Among HCPs to Use the Urgency NRS to Assess Bowel Urgency in Clinical Practice

|  | GI  **(n = 16)** | FM/IM/PCP  **(n = 12)** | PA  **(n = 5)** | NP  **(n = 5)** | Overall  **(N = 38)** |
| --- | --- | --- | --- | --- | --- |
| n (%) |  |  |  |  |  |
| Does not routinely assess  bowel urgency | 0 | 0 | 0 | 1 (20.0) | 1 (2.6) |
| Does not want to add another task to complete during  the patient's appointment | 2 (12.5) | 6 (50.0) | 1 (20.0) | 0 | 9 (23.7) |
| Does not have enough time during an office visit to use this measure | 3 (18.8) | 3 (25.0) | 2 (40.0) | 0 | 8 (21.1) |
| Not familiar with this measure | 4 (25.0) | 7 (58.3) | 2 (40.0) | 2 (40.0) | 15 (39.5) |
| Does not think the measure would be helpful to them | 10 (62.5) | 3 (25.0) | 2 (40.0) | 1 (20.0) | 16 (42.1) |
| The measure is not very applicable for use in their practice | 2 (12.5) | 4 (33.3) | 0 | 1 (20.0) | 7 (18.4) |
| Other | 1 (6.3) | 0 | 0 | 0 | 1 (2.6) |

FM = family medicine physician; GI = gastroenterologist; HCP = healthcare provider; IM = internal medicine physician; NP = nurse practitioner; PA = physician assistant; PCP = primary care physician.

Figure S-1. IBD Symptoms Considered Most Important by Each HCP Type or Specialty When Deciding on a Patient’s Course of Treatment


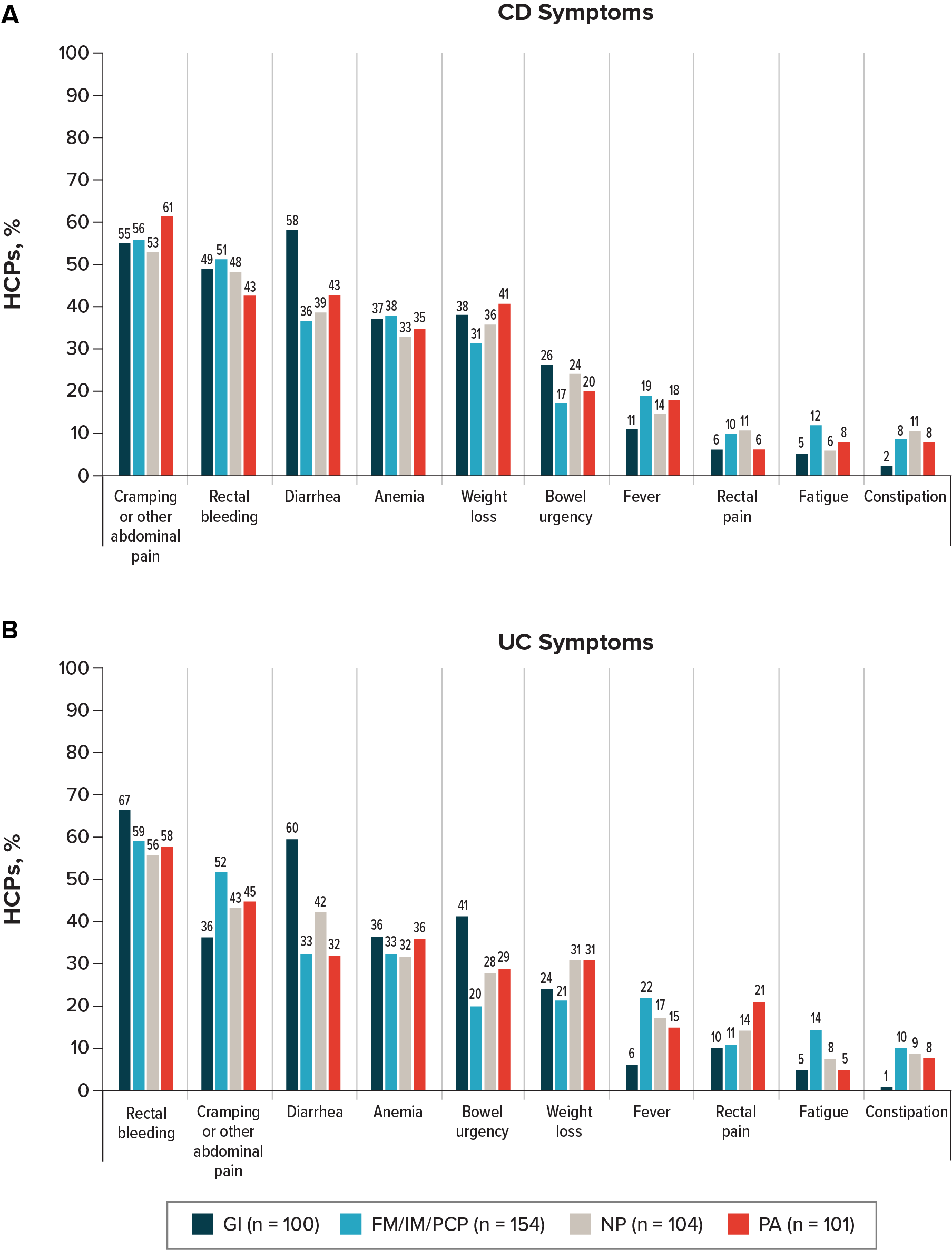


CD = Crohn’s disease; FM = family medicine physician; GI = gastroenterologist; HCP = healthcare provider; IBD = inflammatory bowel disease; IM = internal medicine physician; NP = nurse practitioner; PA = physician assistant; PCP = primary care physician; UC = ulcerative colitis.

Note: For their patients with (A) CD and (B) UC. HCP participants could select up to 3 symptoms. This figure shows the top 10 symptoms. Other CD and UC symptoms included vomiting, loss of appetite, and nausea.

## Figure S-2. Importance of Achieving Complete Disease Control^a^ When Deciding on a Patient’s Course of Treatment

**
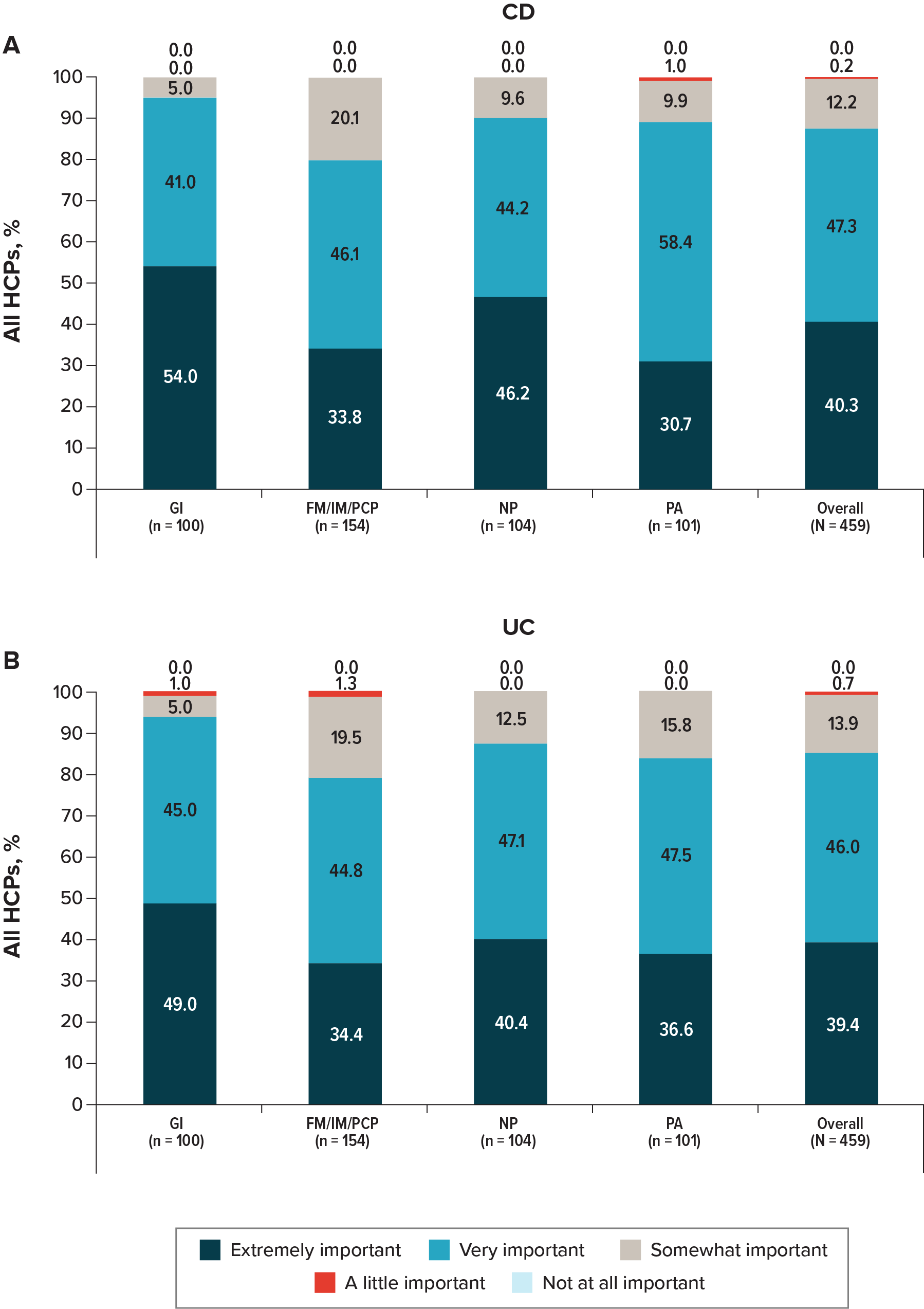
**

CD = Crohn’s disease; FM = family medicine physician; GI = gastroenterologist; HCP = healthcare provider; IM = internal medicine physician; NP = nurse practitioner; PA = physician assistant; PCP = primary care physician; UC = ulcerative colitis.

Note: For patients with (A) CD and (B) UC.

^a^Complete disease control was defined as clinical remission, no bowel urgency, and mucosal/histologic healing.

## Figure S-3. Importance of Not Achieving Complete Disease Control^a^ When Deciding to Escalate the Dose of a Patient’s Current Treatment


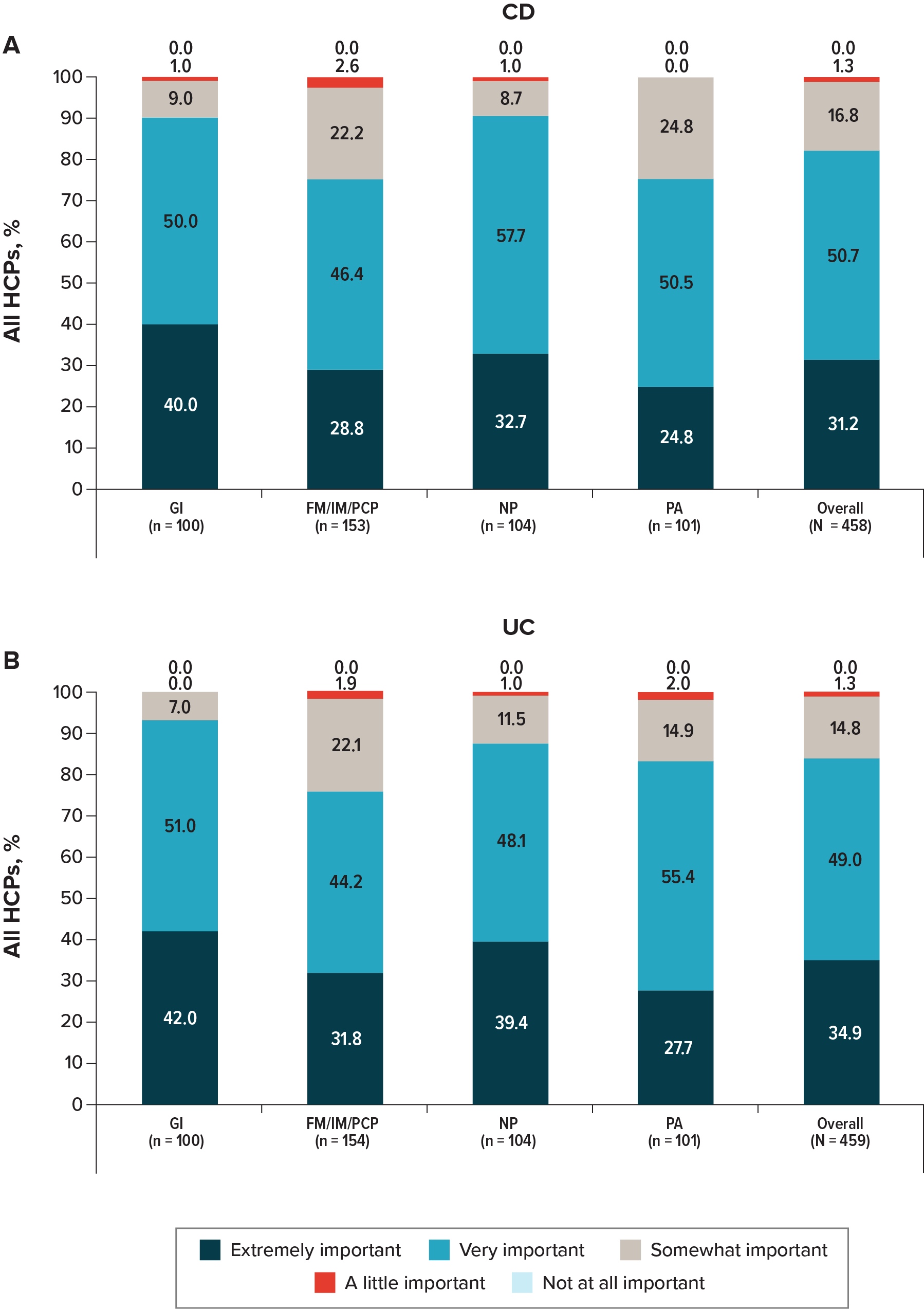


CD = Crohn’s disease; FM = family medicine physician; GI = gastroenterologist; HCP = healthcare provider; IM = internal medicine physician; NP = nurse practitioner; PA = physician assistant; PCP = primary care physician; UC = ulcerative colitis.

Note: For patients with A) CD and B) UC.

^a^Complete disease control was defined as clinical remission, no bowel urgency, and mucosal/histologic healing.

## Figure S-4. Importance of Not Achieving Complete Disease Control^a^ When Deciding to Switch a Patient’s Current Treatment


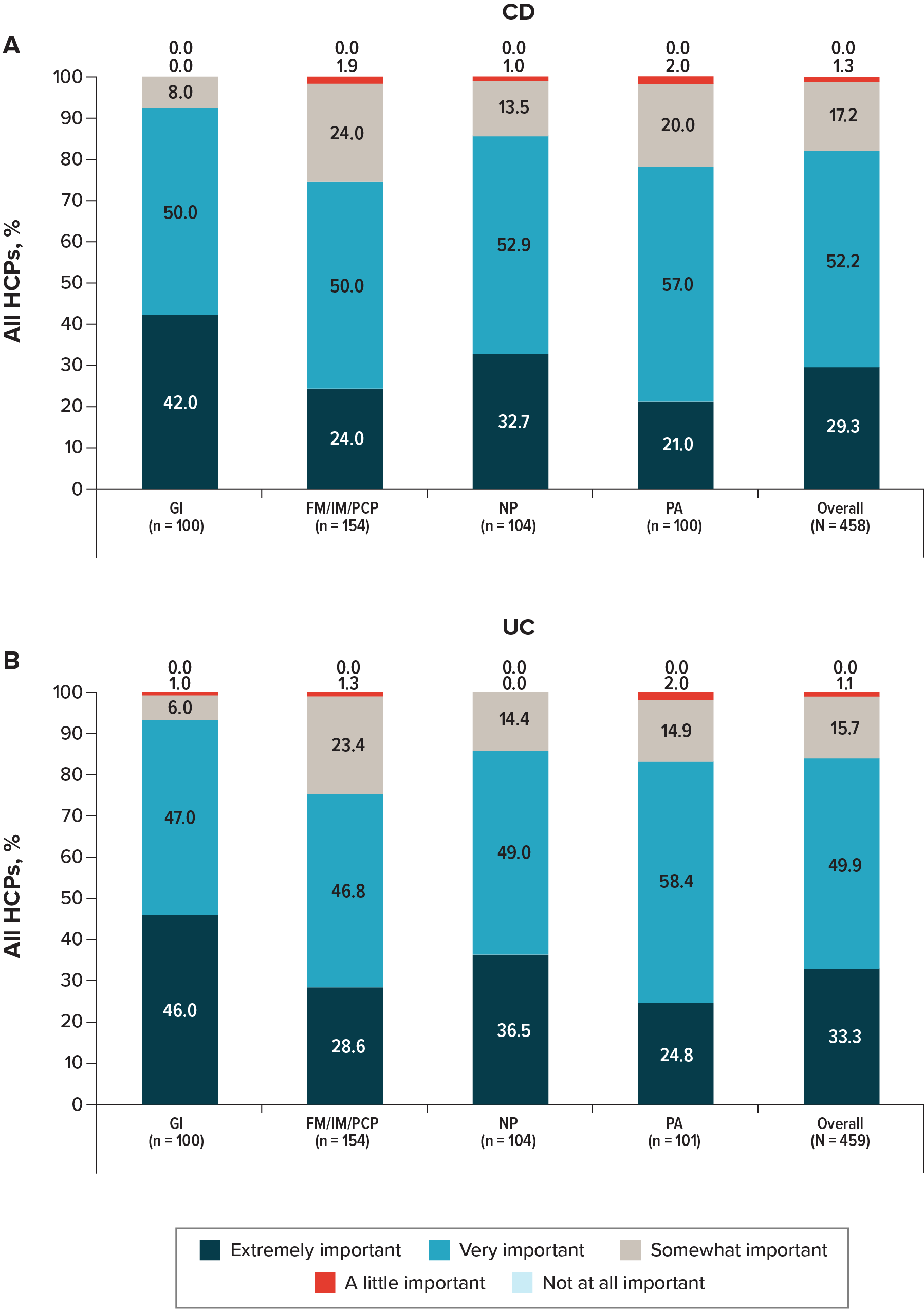


CD = Crohn’s disease; FM = family medicine physician; GI = gastroenterologist; HCP = healthcare provider; IM = internal medicine physician; NP = nurse practitioner; PA = physician assistant; PCP = primary care physician; UC = ulcerative colitis.

Note: For patients with A) CD and B) UC.

^a^Complete disease control was defined as clinical remission, no bowel urgency, and mucosal/histologic healing.

## Figure S-5. HCPs’ Willingness to Use the Urgency Numeric Rating Scale to Assess Bowel Urgency for their Patients with IBD


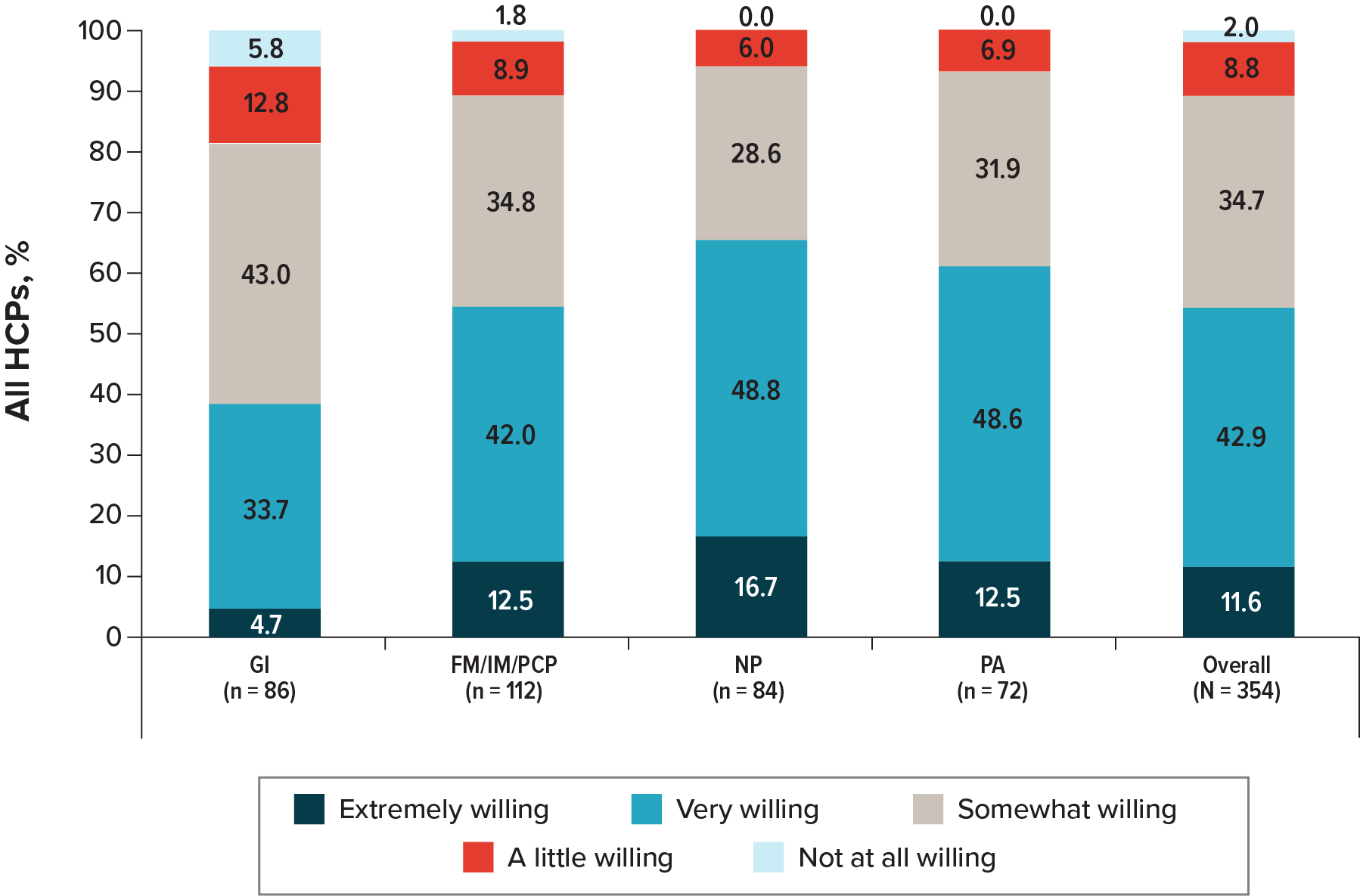


FM = family medicine physician; GI = gastroenterologist; HCP = healthcare provider; IM = internal medicine physician; NP = nurse practitioner; PA = physician assistant; PCP = primary care physician.
